# Are Participants Aware of the Type and Intensity of Transcranial Direct Current Stimulation?

**DOI:** 10.1371/journal.pone.0148825

**Published:** 2016-02-10

**Authors:** Matthew F. Tang, Geoffrey R. Hammond, David R. Badcock

**Affiliations:** 1 School of Psychology, The University of Western Australia, Crawley, WA, Australia; 2 Queensland Brain Institute, The University of Queensland, St Lucia, QLD, Australia; University of California, Merced, UNITED STATES

## Abstract

Transcranial direct current stimulation (tDCS) is commonly used to alter cortical excitability but no experimental study has yet determined whether human participants are able to distinguish between the different types (anodal, cathodal, and sham) of stimulation. If they can then they are not blind to experimental conditions. We determined whether participants could identify different types of stimulation (anodal, cathodal, and sham) and current strengths after experiencing the sensations of stimulation during current onset and offset (which are associated with the most intense sensations) in Experiment 1 and also with a prolonged period of stimulation in Experiment 2. We first familiarized participants with anodal, cathodal, and sham stimulation at both 1 and 2 mA over either primary motor or visual cortex while their sensitivity to small changes in visual stimuli was assessed. The different stimulation types were then applied for a short (Experiment 1) or extended (Experiment 2) period with participants indicating the type and strength of the stimulation on the basis of the evoked sensations. Participants were able to identify the intensity of stimulation with shorter, but not longer periods, of stimulation at better than chance levels but identification of the different stimulation types was at chance levels. This result suggests that even after exposing participants to stimulation, and ensuring they are fully aware of the existence of a sham condition, they are unable to identify the type of stimulation from transient changes in stimulation intensity or from more prolonged stimulation. Thus participants are able to identify intensity of stimulation but not the type of stimulation.

## Introduction

Transcranial direct current stimulation (tDCS) is a widely-used non-invasive technique to manipulate cortical excitability in humans, with anodal stimulation increasing and cathodal stimulation decreasing cortical excitability by altering the resting membrane potential of stimulated neurons [1–3]. The potential of tDCS as an experimental and therapeutic tool has been demonstrated through its ability to enhance motor learning [4], reduce high-level visual distraction [5], dissociate auditory processing mechanisms [6], and reduce depressive symptoms [7], in addition to numerous other effects reported in the last 10 years [8]. Its usefulness as an experimental tool, however, depends partly on whether participants are effectively ‘blind’ to stimulation conditions, given by the polarity and type of stimulation (anodal, cathodal, and the typical control sham stimulation condition) and its intensity.

There are a number of sensations commonly-reported during stimulation that may allow for participants to identify the stimulation condition. Most participants describe a mild itching, tingling, or burning sensation when the current is initially applied. Stimulation can induce more serious effects such as headaches and mild pain in a small minority [9, 10]. Sham stimulation, where current is ramped up and down briefly (generally over 20–30 s) at the beginning of the procedure, is commonly used for experimental control, allowing participants to experience the sensations associated with current onset or offset while avoiding cortical excitability changes from sustained stimulation. A number of studies have examined whether participants are able to identify whether they received active or sham stimulation through differences in evoked sensations [9–15]. These studies have focused on whether ramping the current up and down, or extended periods of constant-current stimulation, allows for placebo control, an important issue for controlling expectation effects for effective placebo control. Most of these studies, using a 1-mA current, found more sensations were reported with active (either anodal or cathodal) than sham stimulation. However, when questioned, participants could not identify whether the stimulation was active or sham [9, 14]. One study [12] claimed that type of stimulation could not be blinded at 2 mA, contradicting another report that blinding is possible at this intensity [13].

No study, to our knowledge, has determined whether participants can identify the type of stimulation or different current intensities [16], an important issue for within-subject designs where participants may be exposed to different stimulation conditions in different sessions. Furthermore, we aimed to clarify whether sham stimulation can be distinguished from active stimulation at higher current intensities, as there have been a number of recent conflicting results about this issue [12, 13]. To this end, we examined whether stimulation type (anodal, cathodal, sham) can be identified at a higher (2 mA) and lower (1 mA) intensities. These questions were addressed by familiarizing participants with the sensations evoked by the different stimulation types, at each of two current intensities, then asking them to identify the stimulation type and current intensity.

Experiment 1 focused on whether the sensations experienced during the initial period of stimulation are different between stimulation types, as the strongest sensations of stimulation occur during this time [9, 10, 17]. This brief stimulation period allowed us to expose each participant to each of the six stimulation conditions (anodal, cathodal and sham, each at 1 and 2 mA) twice. Experiment 2 used a similar design but increased stimulation duration closer to that used in typical experiments with participants. For both experiments, it was hypothesized that participants would not be able to identify the type of stimulation (anodal, cathodal or sham) at either current intensity. It was also hypothesized that participants would be able to identify the current intensity (1 or 2 mA), as higher current intensity is associated with stronger sensations.

## Experiment 1

### Methods

#### Participants

Sixteen adults (10 females) aged between 17 and 47 yr (median age = 18.5 yr) were recruited from an undergraduate psychology pool. This sample size was appropriate for assessing the sensations in an experimental context as it is similar to previous experimental tDCS studies that used within-subjects designs [2, 6, 18]. There were eight participants in each stimulation site (M1 or V1) group. Participants were not recruited if they reported any conditions contraindicative to the use of tDCS as assessed through a checklist used during recruitment [10]. No participants reported any adverse effects of stimulation, apart from a reddening of the skin under the electrodes, and none withdrew from the study. All participants had normal or corrected-to-normal acuity as assessed with a LogMAR chart.

#### Ethics statement

The procedure was in accordance with the principles established by the Declaration of Helsinki and the procedure was approved by the Human Research Ethics Committee at the University of Western Australia. All participants provided written, informed consent prior to testing. Three participants were 17 years old at the time of testing (turning 18 that calendar year), but in-line with ethical approval provided their own written consent. The ethics board was aware of the recruitment procedures.

#### tDCS procedure

tDCS was delivered by a constant-current generator (Dupel Iontophoresis System, MN) through two 6 × 4 cm (24 cm^2^) saline-soaked electrodes in pouches on the scalp. Separate groups of participants received stimulation to either M1/contralateral supraorbital area, as it is likely the most widely-used stimulation montage in experimental studies e.g. [1, 2, 19], or V1/Cz, because tDCS is increasingly being used in visual perception studies e.g. [20, 21–26]. For the M1 stimulation group, the active electrode was placed on C3 in the International 10–20 system and the reference was placed above the contralateral orbit. The active electrode was placed directly above the mastoid bone and the reference was placed on Cz for the V1 stimulation group.

The stimulation for anodal and cathodal conditions was initially ramped on over ~30 s then maintained at the specified level (1 or 2 mA) for 30 s, then ramped off over ~25 s (total stimulation time 85 s). The current density was 0.042 mA/cm^2^ for the 1 mA condition and 0.083 mA/cm^2^ for the 2 mA condition. Stimulation for the sham condition was initially ramped on to the specified level over ~30 s and then immediately ramped off over ~25 s (total stimulation time 55 s). The electrode arrangement was randomized between anodal and cathodal for the sham stimulation condition.

#### Apparatus

Testing was conducted in a dark room (< 1cd/m^2^) using MATLAB 7.14 and PsychToolbox [27, 28] for stimulus presentation with a Cambridge Research Systems Bit# used to achieve 14-bit gray-scale resolution. A Sony Trinitron G520 monitor, with a resolution of 1024 × 768 pixels and 120 Hz refresh rate, was used for presentation. Viewing distance was 70 cm, maintained using a chinrest, making the screen extend 31° × 23° and thus each pixel extended 1.8’ × 1.8’. The luminance of the monitor was gamma-corrected using a Cambridge Research Systems ColorCAL II and custom-written software [29, 30]. The maximum achievable luminance was 160 cd/m^2^ and background luminance was set at 90 cd/m^2^.

#### Procedure

We assessed whether the sensations associated with tDCS stimulation onset and offset allowed participants to identify stimulation type (anodal, cathodal, or sham) and current intensity (1 or 2 mA) after they had been familiarized with all the stimulation types and intensities. Two groups of participants were tested, with stimulation applied over either M1 or V1, while they completed a visual contrast-rating task that assessed their ability to identify small differences in stimuli. This experiment consisted of familiarization with tDCS, with each type (anodal, cathodal, sham) and current strength (1 and 2 mA) combination presented once after informing the participants of the stimulation type and intensity. This was followed by 12 experimental trials to determine whether participants could identify the stimulation type and intensity (with each combination of type and intensity presented twice in a pseudo-randomized order) while engaged in the contrast-rating task. The participants were informed prior to the familiarization trials that they would be required to identify the type and current intensity of tDCS during the experiment.

An identical contrast-rating task was completed while stimulation was being applied during both familiarization and experimental trials. This task was included for four reasons: first, to mirror common tDCS experimental procedures where participants complete a behavioral task during stimulation i.e. [4, 19]; second, to assess the participants’ sensitivity to small changes in visual stimuli; third, to determine whether this ability is affected by stimulation; and forth, to determine whether tDCS-induced changes in visual performance allow participants to distinguish between the different conditions, in addition to the other types of evoked sensations.

**Fig 1:**
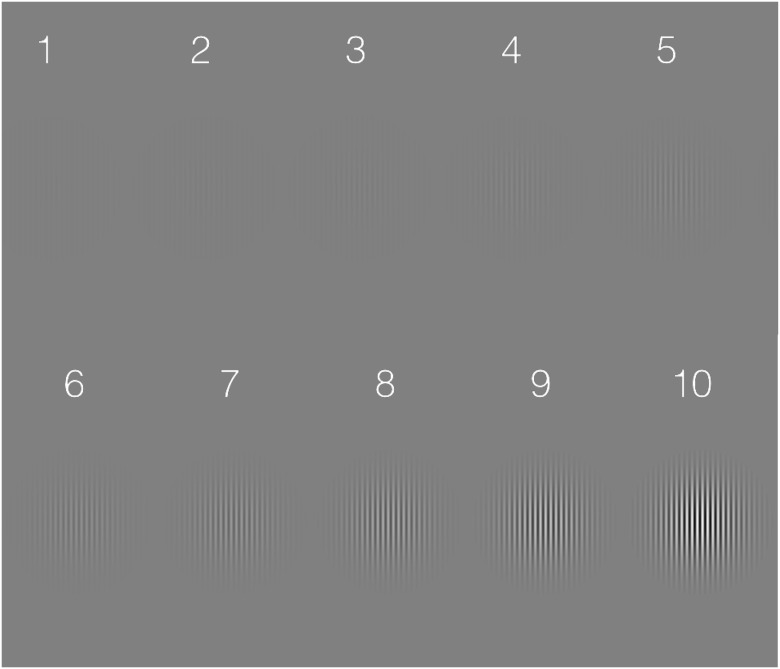
Examples of the 10 Gabor patches used in the contrast-rating task in Experiment 1. On a gamma-corrected monitor, the contrast of these stimuli will appear to increase in logarithmically-spaced steps between 1% (1) and 100% (10). In a block of the task during Experiment 1, each Gabor was presented once in a pseudo-randomized order and the participants rated where it fell on the 1–10 scale. Note the stimuli are not drawn to scale for a reading distance but the relative size of the envelope and carrier are correct.

During the contrast-rating task, 10 targets (Gabor patches) were individually presented with the contrast of the targets varied in logarithmically-spaced steps between 1 and 100% (Fig 1) and the participants rated its contrast on the 10-point scale. The order of presentation of the different contrasts was pseudo-randomized on each trial. The Gabor patches (sinusoidally-modulated gratings enveloped by a two-dimensional Gaussian luminance profile) were 10° in diameter, with a spatial frequency of 4 c/° and standard deviation of 1.67°, and were presented for 500 ms. The temporal luminance profile of the Gabor followed a square-wave function.

**Fig 2:**
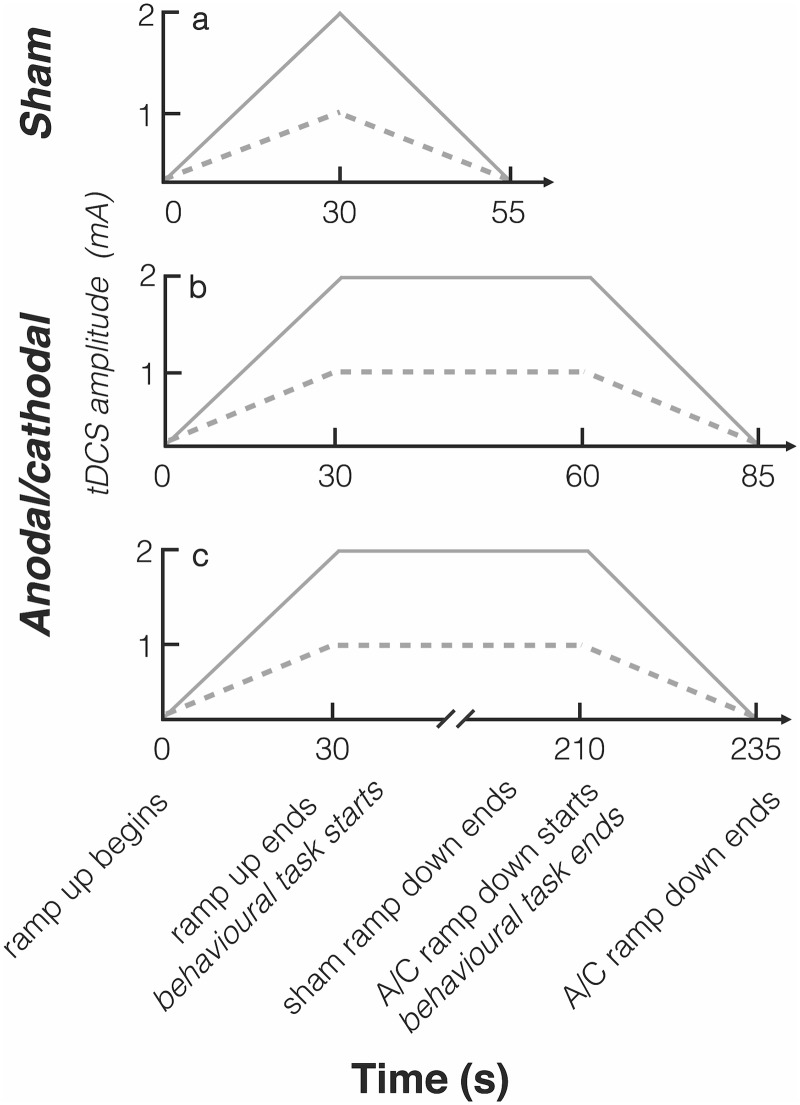
A schematic illustration of the current intensities for the different stimulation types over time. The same sham condition (a) was used in both Experiments 1 and 2. The dotted line indicates the 1 mA current intensity and the solid line indicates the 2 mA current intensity trials. The time course differed for the active conditions between Experiment1 (b) and Experiment 2 (c). The behavioral tasks begun after the end of the ramp up period for both active and sham stimulation types and lasted ~30 s.

The contrast-rating task began 30 s after the onset of stimulation, with the task lasting less than 30 s (see Fig 2). In the 12 identification trials, the participants were additionally required to identify the stimulation type (anodal, cathodal or sham) and current intensity (1 or 2 mA) of stimulation after the stimulation had ramped down. Responses were made using a keyboard prompted by on-screen questions. The total experiment lasted ~30 min.

#### Data analysis

The proportion of correct responses for identifying the stimulation type and current intensity in each condition was separately calculated. Normality-tests were conducted on these data to determine whether they met the assumptions for parametric analyses. One-sample t-tests were used to determine whether these values differed from chance performance for variables that met the assumptions. Otherwise the non-parametric equivalent Wilcoxon signed-rank tests were used. These were planned analyses, as they address the key experimental question whether participants can determine stimulation type and current intensity. A significant result would imply that participants could reliably determine the stimulation condition.

For the contrast-rating task responses, each participant’s mean rating (1–10) for each contrast value was taken for each stimulation condition. Semi-log functions (as the contrast increased in log steps) were fitted to these scores to determine sensitivity. A 2 (Site group: M1, V1) x 3 (Type condition: anodal, cathodal, sham) × 2 (Strength condition: 1, 2 mA) mixed-design ANOVA was used to determine whether stimulation affected perceptual sensitivity.

### Results

Our main question for Experiment 1 was whether participants could use the sensations associated with tDCS current onset and offset to determine the stimulation type and intensity. Experiment 1 explicitly focused on the sensations associated with current on and off phase as these appear critical for the perception, with most sensations associated with stimulation occurring during this period [9, 10, 17]. This short period also allowed us to expose all participants to all stimulation types and current intensities within one session, allowing for more powerful comparisons. Furthermore, this is likely a pertinent interval as current is generally only transiently applied for 5–30 s before the current is ramped to off for sham stimulation [3, 6, 22].

**Fig 3:**
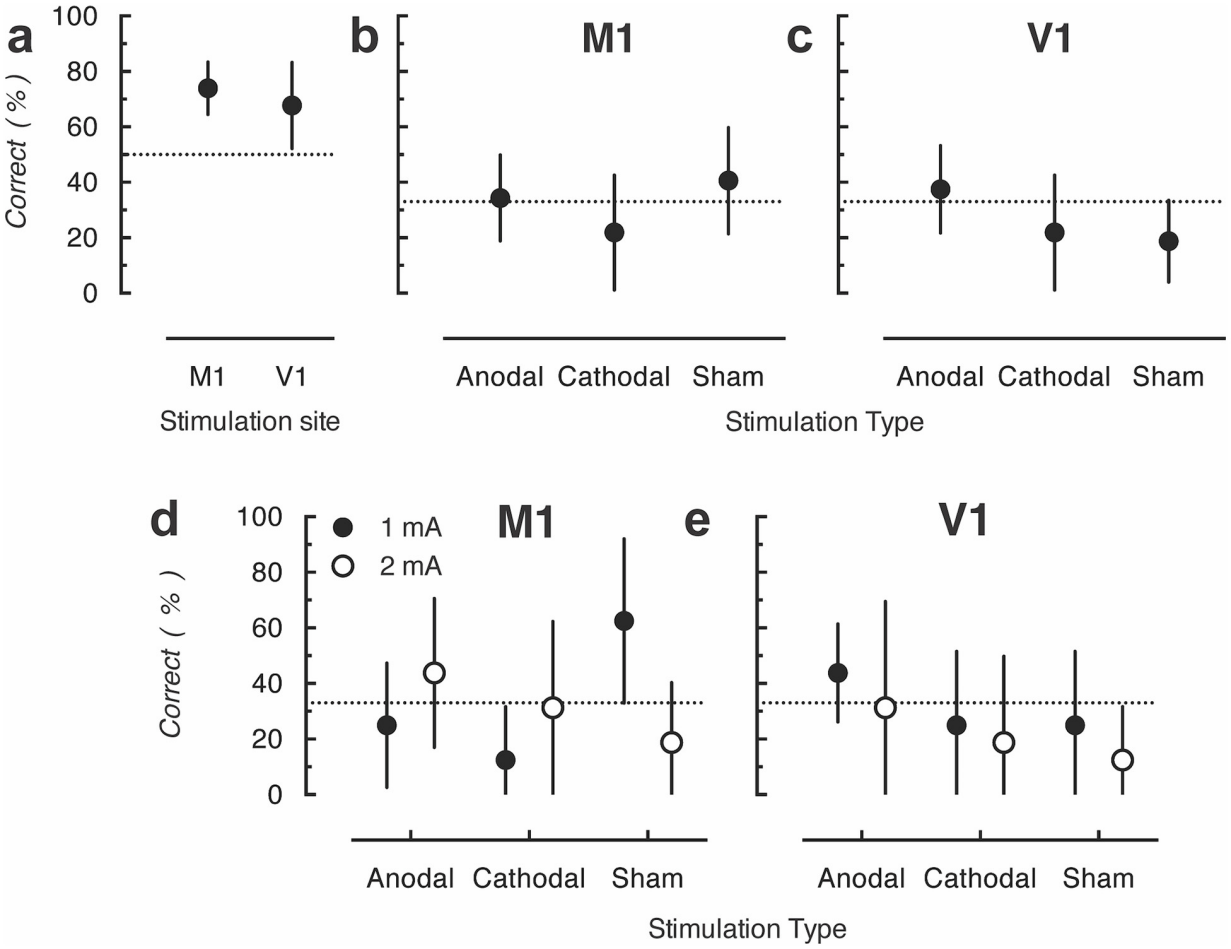
(a) Mean accuracy of judgments of strength of stimulation delivered in the M1 and V1 groups in Experiment 1. (b) Mean accuracy of judgments of type of stimulation delivered. (c) Accuracy of judgments of type of stimulation delivered at the 1- and 2-mA current intensities. The dotted lines indicate the guessing rate for the respective tasks. Error bars represent 95% confidence intervals.

At the conclusion of each trial, participants were asked to respond whether the stimulation type was anodal, cathodal, or sham and whether the intensity was 1 or 2 mA. Both the group receiving stimulation over M1 and the group receiving stimulation over V1 identified current intensity better than chance (Fig 3a); for both groups the 95% confidence intervals did not include the 50% chance level. Planned one-sample t-test confirmed that accuracy significantly exceeded chance (50%) levels for both the V1 (*t*(7) = 2.69, *p* = .03, *d* = 2.03) and M1 (*t*(7) = 6.00, *p* = .0005, *d* = 4.54) stimulation groups.

In contrast, neither group was able to identify stimulation type (Fig 3b), as the percentage of correct responses in all conditions was similar to the chance level (33%). Planned statistical tests showed accuracy did not differ significantly from chance in any condition (all *ps* > .05). The participants’ inability to identify the stimulation type was shown by 95% confidence intervals for identification including the 33% chance level. However, it is possible stimulation types would become discernible at the higher current intensity, as claimed by O’Connell, Cossar (12), as participants could differentiate the two current strengths. The participants’ ability to correctly identify the stimulation type was unaffected by current intensity as the identification rates, as shown by the 95% confidence interval, did not exceed the chance level for either intensity (Fig 3c).

To further break down these results, we looked at whether any individual participant consistently performed better than chance by examining subjects who responded correctly in both trials of any of the six stimulation and current intensity conditions. Four out of the five participants who met this criterion did so in only one condition, with the remaining participant achieving the result in two conditions. Furthermore, these participants were distributed relatively evenly between the groups; with three in the M1 and two were in the V1 stimulation groups. This suggests that their good performance in one condition was due to two correct guesses in a condition rather than an ability to identify the stimulation condition, which would manifest itself as superior performance in all stimulation conditions. These individual results also suggest it is unlikely there are consistent individual differences, such as different sensitivity to stimulation, cortical folding, and impedance between the electrode and scalp, that could systematically affect the ability to identify between the different stimulation types. Therefore, these five participants likely successfully guessed in one condition but were not consistently good identifiers in all conditions.

**Fig 4:**
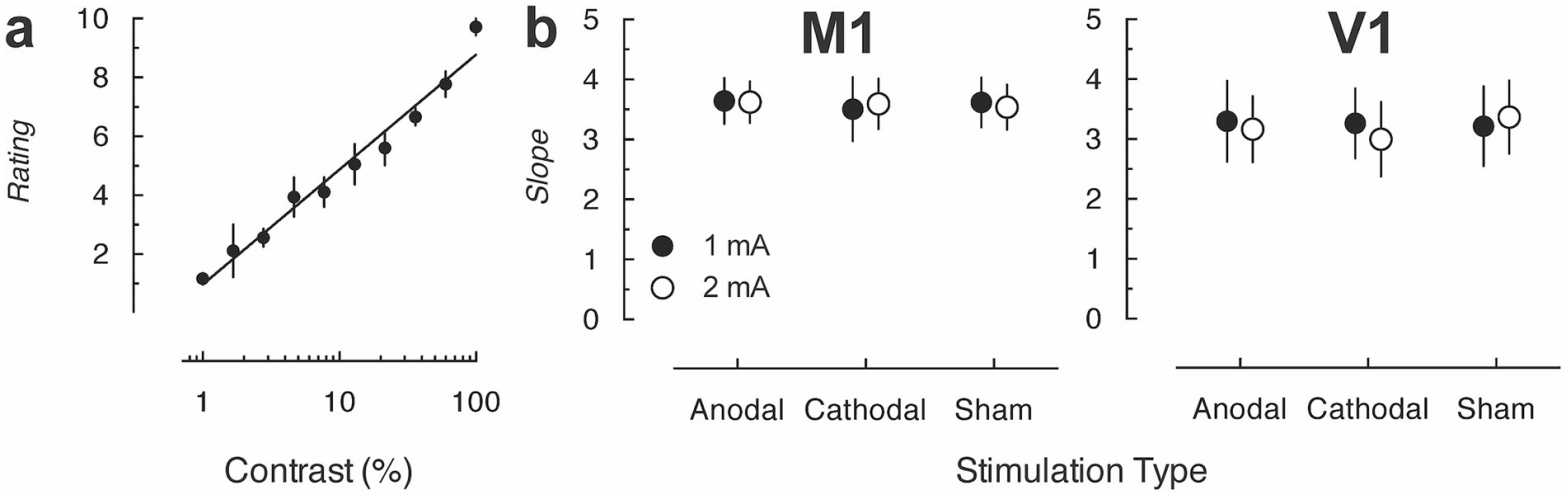
(a) A representative psychometric function in the contrast-rating task in Experiment 1 for one participant fitted with a semi-log line. (b) Mean slope for the fitted semi-log functions for both M1 and V1 stimulation sites. Participants received all stimulation types at different current strengths but not stimulation to both sites. Error bars represent 95% confidence intervals.

We next determined the participants’ sensitivity to small changes in visual stimuli using the contrast-rating task. Responses for the contrast-rating task were fitted with a semi-log function (Fig 4a), the slope of which indicates sensitivity to stimulus change. All participants were able to identify between small changes in the stimuli in the rating task, with ratings correlating strongly with actual contrast levels, mean *R*^*2*^ = .81, *SD =* 6.14. The participants’ ability to make these judgments about small changes in stimuli was unaffected by stimulation over either M1 or V1 as evidenced by the similarity of the slopes in all stimulation conditions (Fig 4b).

A 2 (Site: M1, V1) × 3 (Type: Anodal, Cathodal, Sham) × 2 (Strength: 1, 2 mA) mixed-design ANOVA on the fitted slopes showed that the ability to make these judgments was unaffected by stimulation (all *ps* > .05) and that there were no visual perceptual cues that allowed discrimination of stimulation type in this task that could potentially affect the ability for stimulation blinding, meaning that changes in visual sensitivity could not contribute to the ability to identify the condition.

Taken together, these results strongly suggest that participants cannot differentiate between anodal, cathodal and sham tDCS at either 1 or 2 mA when stimulation is briefly applied, with 30 s of constant current. However, experiments using tDCS typically employ an extended period of constant current stimulation e.g. [1, 18, 19, 20, 21, 25]. It is, therefore, possible that with longer periods of stimulation participants may be able to differentiate the types of stimulation. The next experiment investigated whether participants could identify stimulation type after a more extended stimulation period.

## Experiment 2

### Method

#### Participants

A new group of eight (2 female) participants, between the ages of 18 and 51 yr (median = 21 yr) were recruited from the same undergraduate psychology pool as the initial experiment or through word of mouth. The exclusion criteria were the same as those used in Experiment 1. This sample size was chosen because only one stimulation location (V1) was used, and, therefore, the same power was achieved. No participants reported any adverse effects of stimulation, apart from a reddening of the skin under the electrodes, and none withdrew from the study.

#### Ethics Statement

The same conditions applied as in Experiment 1.

#### Apparatus

The same testing apparatus was used as in Experiment 1.

#### Procedure

The same procedure was used as Experiment 1, expect where noted. The main change was that a constant current intensity was applied for 3 min (compared to 30 s) in the anodal and cathodal conditions. The same stimulation ramp times as in Experiment 1 were used for all conditions. Stimulation was only delivered over V1 as both groups showed similar effects in Experiment 1 (Fig 2). The behavioral task was also changed from a contrast rating to a center surround inhibition task [26, 31]. The task was changed as it took approximately 4 min to complete, so that, as in Experiment 1, the participants performed a task for the duration of simulation. The results from the visual task are not reported because Experiment 1 established that tDCS over V1 does not produce perceptual differences between different stimulation conditions. The experiment lasted ~45 min.

#### Data analysis

The same methods were used as in Experiment 1.

### Results

At the conclusion of each trial, participants were asked to attempt to identify both the type and current intensity of the stimulation. Participants were able to identify the intensity of stimulation on 62.50% of trials, slightly exceeding chance levels (50%). However, unlike in Experiment 1, a Wilcoxon-signed rank test indicated this was not significant, *Z* = 10, *p* = .13, even though there was identical statistical power. The different results between Experiments 1 and 2 were not due to participants being worse at differentiating the current intensity during sham trials where there was a longer period between stimulation ending and participants being asked to identify the intensity, unlike active conditions where stimulation finished just before identification. When sham trials were excluded from analysis, accuracy increased slightly to 65.63% but was still not significantly greater than chance, *Z* = 6, *p* = .25.

**Fig 5:**
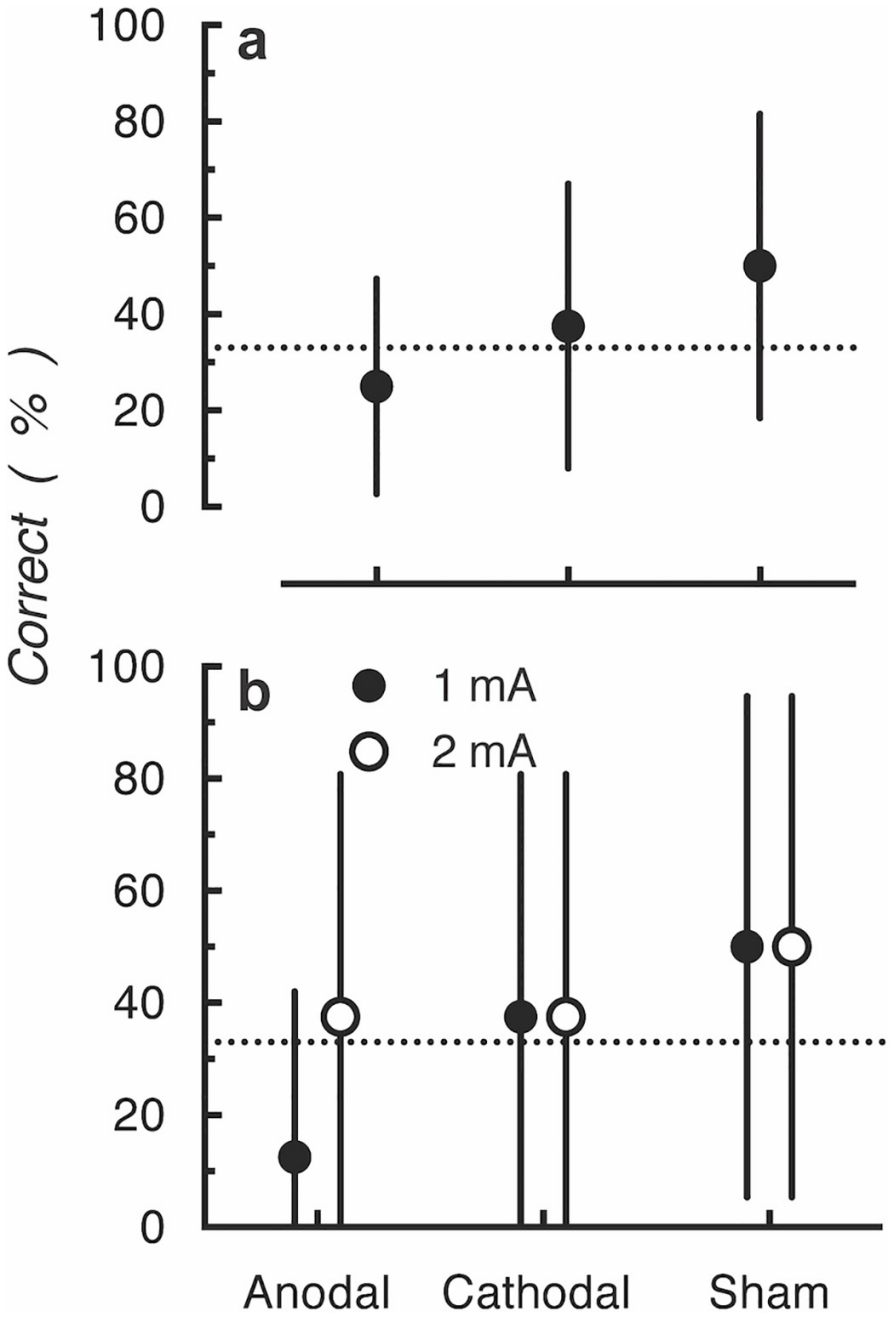
(a) Mean accuracy of judgments of type of stimulation delivered in Experiment 2. (b) Mean accuracy of judgments of type of stimulation delivered at 1 and 2 mA of current intensity. The dotted lines indicate the guessing rate (33%). Error bars represent 95% confidence intervals.

However, replicating the results from Experiment 1, participants were unable to identify the type of stimulation (anodal, cathodal or sham) at better than chance levels (Fig 5a). This is indicated by all 95% confidence intervals overlapping with the chance levels and the statistical tests being not significantly different to chance (all *ps* > .05). Finally, we again sought to determine whether some participants were better able to identify the stimulation type at higher current intensities (Fig 5b). To do this, we separately analyzed identification rates for stimulation type for trials with 1 and 2 mA. The figure shows the 95% of confidence interval always overlapped with the guessing rate at both current intensities.

These results extend the effects reported in Experiment 1, by showing that participants could not identify the tDCS type at either 1 or 2 mA even with an extended (3 min) period of constant current stimulation.

## General Discussion

The present study determined whether participants could identify the intensity and type of tDCS using the skin sensations evoked during stimulation onset and offset (Experiment 1) or during an extended period of constant current (Experiment 2). These are important questions as being able to identify stimulation type could potentially produce differential responses between stimulation groups, independent of the physiological effect of stimulation on neural function. We found that participants could not distinguish between anodal, cathodal and sham stimulation delivered at either 1 or 2 mA even though they were familiarized with the sensations and were able to identify the strength of stimulation with shorter, but not longer, periods of stimulation.

Experiment 1 focused on the initial transient period of stimulation during current on and offset where the sensations associated with stimulation are most noticeable [9, 10, 17]. This brief stimulation period was used as participants generally report that the sensation of stimulation fades when current is maintained at a constant level [9]. Experiment 2 was conducted to determine whether participants could identify stimulation type with longer periods of stimulation. In both experiments, participants were asked to identify the type (anodal, cathodal and sham) and intensity of stimulation (1 or 2 mA). Participants were not able to identify the type of stimulation in either experiment. However, participants were able to identify stimulation intensity at better than chance levels with the brief stimulation duration in Experiment 1 but not with the more prolonged stimulation duration in Experiment 2.

A strength of the current study design was that, unlike most studies, the participants were fully aware of existence of the sham condition, yet could not identify the stimulation type associated with the evoked sensations even after prior exposure. They could, however, reliably identify tDCS intensity with the shorter stimulation durations. These two latter results suggest that participants were sufficiently sensitive to detect small changes in stimuli, yet were unable to distinguish stimulation types. Overall, these results provide an important step in understanding the experience of participants to the sensations associated with tDCS.

Our result showing that participants are unable to identify whether stimulation was active or sham is consistent with a number of previous studies using a 1-mA intensity [10, 11] and one using a 2-mA intensity [14]. The results do, however, conflict with a recent report showing that participants were able to identify active and sham stimulation at 2 mA in the second, but not the first, of two tDCS sessions [12]. The authors claimed their result shows that participants cannot be blinded to tDCS type at 2 mA because of the increased sensations associated with higher current intensities. The different result to our study could be because O’Connell, Cossar (12) used briefer current ramp times (5 s) than the current study (30 s). The relatively sudden current onset and offset could have increased the sensations produced by sham stimulation. However, to our knowledge no studies have examined whether shorter ramp times increase sensations.

Furthermore, our results are consistent with a recent study [13] which found that participants were unable to identify active and sham stimulation at 2 mA delivered for between 10 and 20 min when they were explicitly asked. Our result showing that participants could not identify stimulation type at either current intensity, suggests that the results from previous reports [10, 11] of the effectiveness of tDCS blinding at 1 mA are likely to be generalized to higher current intensities. When taken in conjunction with the majority of previous studies, the results suggest that it is likely that participants can be blinded to stimulation type at both 1 and 2 mA, with the possible caveat that longer (~30 second) intensity ramps may be necessary.

The present results also extend the previous findings as none have examined whether participants can identify anodal and cathodal tDCS, nor whether different current strengths can be identified. Previous studies have found there are stronger sensations (i.e. itching, tingling) associated with active than sham stimulation [9, 14]. Our results, however, taken in conjunction with others [11, 13], suggest that participants are unable to use these increased sensations (during stimulation on- and offset) to determine the stimulation type they are receiving. This is a useful finding for the use of tDCS for within-subject experiments where participants will be exposed to all combinations of stimulation type and, possibly, current intensities. With our results suggesting that participants will be unable to use the differences between conditions in the perceived sensations during stimulation on and offset to determine their stimulation type.

A limitation of the current study is that only 24 participants were tested, meaning that small differences could become statistically significant with a larger sample. However, there was no evidence that any of the participants in either experiment could consistently identify stimulation type above than chance levels. Furthermore, group means were near chance level suggesting that the lack of statistical significance was not due to insufficient power but rather that the evoked sensations of stimulation were indiscernible to participants in the different stimulation types.

Further mention should also be given to the difference in stimulation duration between the active and sham conditions in the current study. The total stimulation duration, including ramp times, was longer (85 s in Experiment 1, 235 s in Experiment 2) for active conditions than the sham condition (55 s in both experiments). The participants were not able to use these differences in duration to determine the stimulation condition, suggesting that the sensations associated with stimulation are most notable only during stimulation onset, not during periods of constant current. Furthermore, strong evoked sensations are likely to be only present during stimulation on- and off-set otherwise the difference in total duration between active and sham conditions would have been noticeable especially with longer periods of stimulation.

There are some further differences between the current study and previous results that could potentially limit the generalizability of the findings. The current study used a slightly smaller electrode pad size (24 cm^2^) than some previous studies that assessed the efficacy of sham stimulation for participant blinding, which used 25 cm^2^, or 35 cm^2^ pads, giving a slightly higher current density. However, Russo, Wallace (14) found that a smaller electrode does not increase the ability to identify sham stimulation compared to a larger electrode, suggesting that this difference is unlikely to have affected the results. A further issue to note is that in this experiment only the participants, not the experimenter, were blinded to the stimulation condition. This was done out of necessity as the equipment required that the experimenter was aware of the stimulation type delivered in each trial. However, the experimenter’s awareness of stimulation type did not allow participants to identify stimulation type.

## Conclusions

We have shown that participants could not identify different types of briefly applied tDCS stimulation following familiarization. The results show that participants are unaware of the different types of tDCS with the sensations evoked by sham condition being indistinguishable from anodal and cathodal stimulation at both 1 and 2 mA of current intensity. This is an important addition to the literature as a recent study has claimed that tDCS cannot be blinded at 2 mA [12]. We show that there are no differences during current onset and offset, nor during a period of constant current, that alert the participants of the stimulation type regardless of current intensity, extending results at both 1 [11] and 2 [13] mA. These issues are particularly relevant for using tDCS for within-subjects experimental designs where the same participants are in all conditions.
